# A comparison of blood stream infections with extended spectrum beta-lactamase-producing and non-producing *Klebsiella pneumoniae* in pediatric patients

**DOI:** 10.1186/s13052-017-0398-0

**Published:** 2017-09-12

**Authors:** Sevgen Tanır Basaranoglu, Yasemin Ozsurekci, Kubra Aykac, Eda Karadag Oncel, Asiye Bıcakcigil, Banu Sancak, Ali Bulent Cengiz, Ates Kara, Mehmet Ceyhan

**Affiliations:** 1 Department of Pediatric Infectious Diseases, Hacettepe University Faculty of Medicine, Sıhhıye, Ankara, Turkey; 2 Department of Microbiology, Hacettepe University Faculty of Medicine, Ankara, Turkey

**Keywords:** Extended spectrum β lactamase, *Klebsiella pneumonia*, Blood stream infections, Children

## Abstract

**Background:**

Rapid development and global spread of multidrug resistant *Klebsiella pneumonia* (*K. pneumoniae*) as a major cause of nosocomial infections is really remarkable. The aim of this study was to explore risk factors for health care associated blood stream infections (BSI) caused by ESBL-producing *K. pneumoniae* in children and analyze clinical outcomes.

**Methods:**

A retrospective review of patients younger than 18 years-old with blood stream infection caused by *K. pneumoniae* was performed. Patients with ESBL-producing *K. pneumoniae* isolates were compared with ESBL-non-producing isolates in terms of risk factors, outcome and mortality.

**Results:**

Among 111 *K. pneumoniae* isolates 62% (*n* = 69) were ESBL –producing *K. pneumoniae*. The median total length of hospitalization and median length of stay in hospital before infection was significantly higher in patients with ESBL-producing isolates than ESBL-non-producing. Use of combined antimicrobial treatment was significantly different between ESBL-producing and ESBL-non-producing groups, 75.4% and 24.6%, respectively (*p* = 0.001). Previous aminoglycoside use was higher in cases with ESBL –producing isolates (*p* = 0.001). Logistic regression analysis showed a significant correlation between mortality and use of combined antibiotics (OR 4.22; *p* = 0.01).

**Conclusion:**

ESBL production in *K. pneumoniae* isolates has a significant impact on clinical course of BSIs. Total length of hospitalization, length of hospital stay before infection, prior combined antibiotic use and use of aminoglycosides were significant risk factors for development of ESBL-producing *K. pneumoniae* related BSI.

## Background

Significant number of health care associated infections in adults and children are caused by gram-negative microorganisms [1, 2]. During the last ten years, changes in antibiotic susceptibility profiles of gram-negative microorganisms have been studied because there is an increasing prevalence of infections due to ESBL (Extended spectrum β lactamase) producing *Klebsiella pneumonia* (*K. pneumoniae*). *K. pneumoniae* is an important opportunistic pathogen of children, causing a wide variety of infections including blood stream infections (BSI); approximately 75% of patients of BSIs are healthcare- associated [2–11]. According to data from National Nosocomial Infections Surveillance System in United States in 2003, *K. pneumoniae* accounted for 18% of the gram-negative bacteremia and 4.2% of all bacteremia [12]. Since the treatment strategies of these infections are limited [3], children with these infections tended to experience high rates of mortality and longer lengths of hospitalization after infection compared to children with BSI due to non-ESBL-producing isolates [13]. Although risk factors for bacteremia with ESBL-producer gram-negative bacilli have been studied in many studies for adults, it is still a serious concern for children on account of few pediatric studies. The objectives of this study were to assess risk factors for health care associated BSI caused by ESBL-producing *K. pneumoniae* in children, analyze clinical outcomes and compare with ESBL-non-producers.

## Methods

This retrospective cohort study was performed in Hacettepe University Ihsan Dogramaci Children’s Hospital in Turkey.

### Patient selection

Patients younger than 18 years-old with positive blood cultures for *K. pneumoniae* from January 2011 to December 2015, were involved in the study. Health –care associated blood stream infection was defined as positive blood culture, obtained from a peripheral vein or central catheter, that is collected at least 48 h after hospital admission and within 48 h if the patient was hospitalized within the previous 60 days. Clinical findings of bacteremia were accepted as presence of at least one of fever, chills or hypotension [14].

### Study protocol

Information regarding clinical and demographic characteristics; underlying medical conditions; total length of hospitalization (LOH); length of stay in hospital before infection; duration of treatment for infection; immunosuppression due to chemotherapy induced neutropenia (absolute neutrophil count <500 cells/mm^3^); previous surgical procedure during hospital stay; mechanical ventilation; polymicrobial bacteremia; presence of central venous catheter; prior colonization of urinary tract and/or lower airway and/or catheter colonization with *K. pneumoniae* before identification of BSI; antimicrobial exposure (only the ones for at least 48 h during the previous 14 days were studied): broad spectrum cephalosporins, and β-lactam-β-lactamase inhibitor combinations, aminoglycosides, carbapenems, fluoroquinolones, glycopeptides (vancomycin or teicoplanin), and exposure to combined antibiotics studied. Extended spectrum beta lactamase production was defined as presence of beta-lactamases that hydrolyse penicilins, 1-3rd generation cephalosporins, but do not hydrolyse cefoxitin or carbapenems and are generally inhibited by beta-lactamase inhibitors [15]. Outcome was evaluated as follows 1) Response at day 6 with occurrence of one of the followings: resolution of fever and other local or systemic signs of infection, normal white blood cell count, and negative blood culture. 2) Relapse: recurrence of infection with a positive blood culture within a month after discontinuation of therapy. 3) Mortality was analyzed as 30-day and 60-day mortality [16]. Number of isolates were also evaluated by years in order to investigate variations in time.

### Microbiological analysis

For identification and antimicrobial susceptibility testing of the isolates BD Phoenix (BD Diagnostics System, Sparks, MD) automated system used between January 2010 and June 2013. After June 2013*,* identification of *K. pneumoniae* isolates was done by matrix-assisted laser desorption ionization–time of flight mass spectrometry (MALDI-TOF MS) and antimicrobial susceptibility testing was performed by using VITEK 2 compact (bioMérieux, Marcy-l’Étoile, France) system. The results of antimicrobial susceptibility tests were interpreted according to Clinical and Laboratory standards Institute (CLSI) recommendations [17].

### Statistical analysis

Statistical analyses were performed using IBM SPSS Statistics (Windows, Version 22.0. Armonk, NY: IBM Corp.) Descriptive analyses were performed including medians, interquartile ranges, standard deviations for continuous variables and frequency distributions for categorical variables. *P* values were calculated using the Chi -Square or Fisher exact tests to compare categorical variables and Mann-Whitney U test to compare continuous variables (*p* value ≤0.05). Variables with *p* values <0.20 in univariate analysis were put into multivariate analysis in order to identify independent risk factors. To determine adjust effect of risk factors on mortality logistic regression was used (*p* value ≤0.10 was considered significant). For analysis of factors that lengthen total stay time in hospital linear regression model was performed with a significant *p* value ≤0.05.

## Results

A total of 97 pediatric patients with 111 *K. pneumoniae* isolates were included in the study. Median age of patients was 8.0 months (IQR: 2–45.6). Sixty-nine isolates (62%) were ESBL- producer and 42 (37.8%) were ESBL-non-producer. Twenty-eight (25.2%) of the isolates presented carbapenem-resistance. Most of the patients in ESBL-producing group were males (63.8%). No statistically significant differences were found between the groups in terms of gender (*p* = 0.99), and patients with ESBL-producing isolates were younger than ESBL-non-producers (*p* = 0.04). Patients younger than 12 months of age constituted 57.7% (*n* = 56) of overall group. Underlying medical conditions/diseases were not significantly different between two groups. ESBL- producing group displayed 7 (11.4%) hematologic malignancy, 6 (9.8%) oncologic malignancy, 8 (13.1%) congenital heart diseases, 2 (3.2%) primary immunodeficiencies, 10 (16.3%) neurologic/metabolic diseases, 9 (14.7%) gastrointestinal diseases, 6 (9.8%) premature birth (Table 1).

**Table 1:** Demographic characteristics of patients, risk factors and outcome

	ESBL (+) *n* = 69	ESBL (−) *n* = 42	*p*
Gender^a^			0.99
Male	37(63.8)	21(36.2)	
Female	24(61.5)	15(38.5)	
Age (months)^b^	4.9(1.7–26)	16.4(3.1–69.1)	0.04
Total length of hospitalization (days)^b^	56(32–89)	34(21–71)	0.03
Length of stay in hospital before infection (days)^b^	33(11.5–59.5)	11.5(0–26)	0.001
Duration of treatment for infection (days)^b^	16(11–21.5)	17(12–23)	0.66
Underlying medical condition/disease^a^			NA
Hematologic malignancy	7(11.4)	8(22.2)	
Oncologic malignancy	6(9.8)	10(27.2)	
Congenital heart anomalies	8(13.1)	4(11.1)	
Primary immunodeficiencies	2(3.2)	1(2.7)	
Neurologic/Metabolic disease	10(16.3)	4(11.1)	
Gastrointestinal disease	9(14.7)	6(16.6)	
Prematurity	6(9.8)	1(2.7)	
Others	13(21.3)	2(5.5)	
Mechanical ventilation^a^	31(81.6)	7(18.4)	0.005
Presence of central venous catheter^a^	34(60.7)	22(39.3)	0.90
Prior surgery^a^	33(66)	17(34)	0.57
Polymicrobial bacteremia^a^	6(42.9)	8(57.1)	0.19
Prior chemotherapy associated neutropenia^a^	15(46.9)	17(53.1)	0.058
Prior *K. pneumoniae* colonization^a^	7	5	NA
Prior antibiotic use^a^			
Broad spectrum cephalosporins	21(75)	7(25)	0.16
Fluoroquinolones	15(75)	5(25)	0.29
Carbapenems	25(61)	16(39)	1.0
Aminoglycosides	47(77)	14(23)	0.001
Glycopeptides	35(71.4)	14(28.6)	0.11
Use of more than one of the antibiotics studied^a^	52(75.4)	17(24.6)	0.001
Outcome^a^			
Crude Mortality	20 (29)	7 (16.7)	0.21
30 day mortality	17(24.6)	5 (11.9)	0.16
60 day mortality	19 (27.5)	7(16.7)	0.28
Response at day 6	42(58.3)	30(41.7)	0.44
Relapse	5(4.5)	4(3.6)	0.67

^a^number, %; ^b^Median, IQR: Interquartile range

ESBL (+): ESBL –producing isolates, ESBL (−): ESBL –non-producing isolates

The median total length of hospitalization was significantly different between ESBL-producers (56, IQR:32–89 days) and ESBL-non-producers (34, IQR: 21–71 days) (*p* = 0.03). The median length of stay in hospital before infection was significantly longer for ESBL- producing group than that of ESBL-non-producing group (*p* = 0.001). Duration of treatment for infection displayed no significant difference (Table 1). Of all, 12.6% (*n* = 14) of isolates were polymicrobial. The microorganisms identified in addition to *K. pneumoniae* were *Pseudomonas aeruginosa* (*n* = 5), *Enterobacter cloacae* (*n* = 3), *Enterococcus faecium* (*n* = 2), *Enterococcus faecalis* (*n* = 1), *Candida tropicalis* (*n* = 1), *Candida parapsilosis* (*n* = 1), *Acinetobacter baumanii* (*n* = 1).

Univariate analysis of predisposing factors such as presence of central venous catheter, prior surgery, polymicrobial bacteremia, presence of chemotherapy associated neutropenia and prior *K. pneumoniae* colonization depicted no statistically significant difference whereas presence mechanical ventilation was different between ESBL-producer (81.6%) and ESBL-non-producer (18.4%) groups (*p* = 0.005).

We performed analysis on relationship of previous antibiotic usage before identification of BSI. Use of combined antimicrobial treatment was significantly different between ESBL-producing and ESBL-non-producing groups, 75.4% and 24.6%, respectively (*p* = 0.001). When analysis of antibiotic regimens was considered, previous aminoglycoside use was higher in patients with ESBL-producing isolates than the ones with ESBL-non-producing isolates (*p* = 0.001) (Table 1).

Although mortality, treatment response at day 6 and relapse rates were not significantly different between comparison groups, ratios of crude mortality, 30-day and 60-day mortality were relatively higher for patients with ESBL producing isolates (respectively, 29%, 24.6%, 17.5%, *p* > 0.05). Logistic regression analysis showed a significant correlation between mortality and two variables: mechanical ventilation (OR 2.4; CI 0.933–6.211; *p* = 0.06) (CI: Confidence interval) and use of combined antibiotics (OR 4.22; CI 1.293–13.791; *p* = 0.01). In addition to this, linear regression displayed a significant correlation between total length of hospital stay and three variables: polymicrobial bacteremia (OR 117.148; CI 73.634–160.663; p = 0.0), prior glycoprotein use (OR 41.96; CI 12.697–71.223; *p* = 0.05), presence of central venous catheter (OR 32.970; CI 3.871–62.069; *p* = 0.02) (Table 2).

**Table 2:** Multivariate analysis for mortality and LOH

	OR	95% CI	*p*
Mortality related risk factors			
Mechanical ventilation	2.4	0.933–6.211	0.069
Use of combined antibiotics	4.22	1.293–13.791	0.017
Risk factors that lengthen total stay in hospital (days)			
Polymicrobial bacteremia	117.148	73.634–160.663	0.00
Prior glycopeptide use	41.96	12.697–71.223	0.05
Presence of central venous catheter	32.970	3.871–62.069	0.027

*OR* odds ratio, *CI* confidence interval. *LOH* total length of hospitalization

**Fig. 1:**
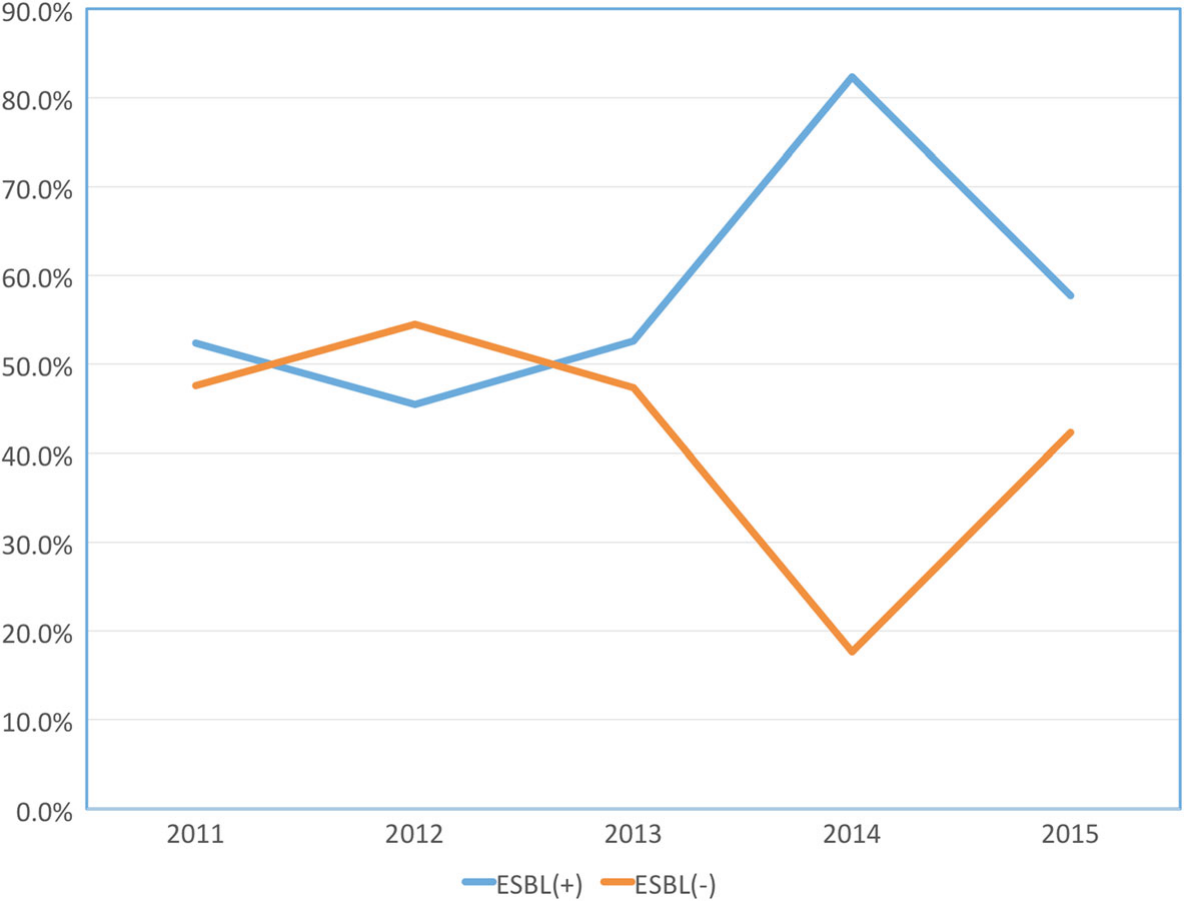
Distribution of *K. pneumoniae* isolates by years

When the distribution of isolates was assessed by year, there was no significant difference between years in univariate analysis in terms of percentage of ESBL-producing isolates. ESBL-producing strains were relatively high in 2014 (*n* = 28, 82.4%) compared to previous 3 years, going forward with a decline in 2015 (*n* = 15, 57.7%) (Fig. 1).

## Discussion

Here, we report clinical significance and outcomes of pediatric health care associated BSIs caused by ESBL-producing and ESBL-non-producing *K. pneumoniae,* over the 5 years study period. In the present study, in 5 years period we observed 62% ESBL production in *K. pneumoniae*, causing BSI. Blood stream infections are one of the most important complications of health care settings. In a multi-centered study of nosocomial infections in pediatric patients, 36% had bacteremia and 37% of patients constituted gram-negative bacilli [2]. Extremely resistant strains are coming into view in several gram-negative microorganisms with resistance to all commonly used antimicrobials [18]. In early reports along Europe, 37.5% of *K. pneumoniae* were ESBL-producer [2]. Felder K et al. reported that ESBL-producing strains ranged from 20.3% to 22.5% [19]. Many other regions worldwide stated a prevalence of ESBL in *Enterobacteriacea spp.* approximately 10–35% [20]. Statistics vary widely from continent to continent and from center to center, with prevalence up to 70% [21]. Our higher result compared to literature may possibly be attributed to our patient spectrum, consisting of many clinically high risky groups including hematology- oncology malignancies, patients in intensive care units and neonates. These groups need a variety of interventions, accompanying long lasting and combined therapies during their hospital stay. Furthermore, analysis of distribution of isolates by years revealed a peak in 2014. We have been strictly following the deviations of these infections in our hospital. According to our remarkable numbers, we introduced some regulatory preventive strategies. The precautions include isolation of the patients with antibiotic resistant microorganisms, follow-up of isolated patients, effective study of committee of hand hygiene including vigorous education of personnel, control of inappropriate broad spectrum antibiotic use, especially including carbapenems.

Levy et al. reported that gram-negative organisms encounter for more than 50% of all bacteremia in children while *K. pneumoniae* accounts for the most commonly identified pathogen, comprising 26% of isolates [6]. In early studies among pediatric BSIs caused by *K. pneumoniae,* 67% were younger than 12 months of age and 93% of the patients had an underlying condition predisposing to opportunistic infections [22].

In the SENTRY report in 2004, ESBL production was reported to be 41.7% in children younger than 1 year of age and 22.5% between 1 and 12 years of age [23]. Other studies revealed a 2-fold decrease in prevalence of *Enterobacteriaceae* (*Klebsiella* spp. and *Enterobacter* spp.) after the age of 1 year [19]. Supporting these data, in the present study, patients younger than 12 months of age constituted 57.7% of overall group and younger patients were significantly under greater risk for BSI with ESBL-producing *K. pneumoniae.*

In the current study, clinical responses were similar for patients with ESBL- producing and non-producing *K. pneumoniae*. Although analysis did not revealed statistical significance, crude mortality, 30-day and 60-day mortality were relatively higher in patients with ESBL-producing isolates. Infection related mortality of the group in the present study (24.6%) was similar to the cohort study of Kim et al. in which 26.7% of children with BSI caused by ESBL (+) bacteria died as compared with 5.7% mortality in patients with non-producers with statistical significance [24]. The results are varying among the centers. In a report from Tanzanian children with septicemia, fatality rate was 71% with ESBL (+) gram-negative bacteria [25].

Many adult studies identified many risk factors for ESBL (+) *K. pneumoniae* infections. Prolonged hospital stay, prolonged stay in intensive care unit, recent exposure to multiple antibiotics and indwelling invasive devices were determined as risk factors for acquisition of ESBL (+) *K. pneumoniae* in adult populations [24, 26–28]. In one of the pediatric studies Kim et al. identified prior hospitalization, prior intensive care unit admission, mechanical ventilation, presence of central venous catheter, development of breakthrough bacteremia during antibiotic therapy, and exposure to extended-spectrum cephalosporins within 30 days of infection as risk factors for blood stream infections caused by ESBL (+) *K. pneumoniae* in neonates with bacteremia [24]. Benner et al. studied the outcome of patients with ESBL producing bacterial infections in pediatric intensive care unit (PICU) [29] and they found that patients with infections due to ESBL-producing *K. pneumoniae* had a longer duration of hospital stay as well as PICU stay, while length of stay in the PICU achieved statistical significance. Analysis of total length of hospitalization revealed a significant difference between ESBL producer and non-producer *K. pneumoniae* isolates in the present study. Further analysis showed that risk factors prolonging total length of hospitalization were presence of accompanying microorganisms to the infection, prior glycopeptide use within last 14 days before infection and presence of central venous catheter during infection. Additively, length of hospital stay before infection was significantly longer for ESBL producers, potentiating the risk of hospital acquisition. A previous pediatric study, similarly, identified the significant association between ESBL (+) *K. pneumoniae* BSIs and longer length of hospital stay before infection [30]. Substantiating these findings, a recent adult study showed significant difference between ESBL-producer and non-producer *Enterobacteriacea*, concerning duration of time from hospital admission to positive blood culture [31].

We found significant association between ESBL-producing *K. pneumoniae* bacteremia and use of combined antibiotics in the study. Among the antibiotics previous aminoglycoside use significantly increased risk of blood stream infection. Until now, for children, there are some studies supporting association of previous antibiotic use and ESBL producing *Enterobacteriacea,* many focusing on urinary tract infections and prophylaxis [32–35]. Recently, Zerr et al. delineated a relative risk of having ESBL producing *E. coli* and *K. pneumoniae* than non-producing isolate 2.2 times higher among antibiotic users in last 30 days of infection. In addition, they stated that subgroup analysis resulted in a significant relation between Amp-C producing *Enterobacteriacea* isolates and third generation cephalosporin use [36]. All of these findings exhibit the close relationship between prior antibiotic exposure and subsequent infections with resistant *K. pneumoniae.*

This study had several limitations. Firstly, due to the retrospective design, data was obtained from clinical reports, so some incomplete data was unavoidable and some characteristics showed no or limited statistical significance. Secondly, the data lacks genotyping and molecular analysis, which if shown, would be very valuable demonstrating our regional pediatric profile.

## Conclusion

As shown in many adult studies, ESBL producing *K. pneumoniae* is one of the major causes of health care associated BSI infections in pediatric patients. We found that total length of hospitalization, length of hospital stay before infection, being younger than 12 months of age, and prior antibiotic use especially aminoglycosides were strongly affecting development of BSI with resistant *K. pneumoniae.* Use of combined antibiotics and mechanical ventilation before infection were increasing risk of mortality due to ESBL-producing *K. pneumoniae* related BSI. It is obvious that some of these factors can be preventable. These results suggest that proper antimicrobial agent selection, appropriate durations of treatment and less invasive procedures may reduce incidence of ESBL-producing *K. pneumoniae* in children*.*

## Abbreviations

BSI: Blood stream infections
CI: Confidence interval
CLSI: Clinical and Laboratory standards Institute
ESBL: Extended spectrum β lactamase
*K. pneumoniae*: *Klebsiella pneumonia*
LOH: Total length of hospitalization
MALDI-TOF MS: Matrix-assisted laser desorption ionization–time of flight mass spectrometry
